# Rapid ventricular pacing in cerebral aneurysm clipping: institutional workflow, systematic review, and single-arm meta-analysis

**DOI:** 10.1007/s10143-025-03668-x

**Published:** 2025-06-11

**Authors:** Johannes Wach, Martin Vychopen, Ferdinand Weber, Felix Arlt, Erdem Güresir

**Affiliations:** Department of Neurosurgery, University Hospital Leipzig, Liebigstr. 20, 04103 Leipzig, Germany

**Keywords:** Cerebral aneurysm, Clipping, Subarachnoid hemorrhage, Rapid ventricular pacing

## Abstract

**Background:**

This study examines the safety and efficacy of rapid ventricular pacing for cerebral aneurysm clipping, focusing on arrhythmia, mortality, aneurysm obliteration, neurological deficits, and myocardial damage assessed via postoperative troponin T levels, through an institutional series, systematic review, and meta-analysis.

**Methods:**

Data were extracted from institutional database and published studies investigating the use of RVP in both ruptured and unruptured aneurysms. Outcomes analyzed included postoperative arrhythmia, mortality, complete obliteration of aneurysms, pacing cycles, mean arterial pressure (MAP) during pacing, pacing rates, and postoperative troponin T levels. Pooled event rates and proportions were calculated using a common effect model, and heterogeneity across studies was assessed using I² statistics.

**Results:**

In 15 institutional cases, RVP-assisted aneurysm clipping achieved stable neurological outcomes, no cardiac complications, and 94% aneurysm obliteration. Combined with literature (141 patients), pooled arrhythmia and mortality rates were 1% and 0%, respectively. Aneurysm obliteration was 92%, new neurological deficits 4%, and troponin T levels 37.7 ng/L. Mean pacing rate, cycles, and MAP were 187.4 bpm, 6.5, and 41.1 mmHg.

**Conclusion:**

The findings suggest that rapid ventricular pacing in cerebral aneurysm clipping is associated with a low risk of cardiac arrhythmia and myocardial injury, while facilitating high rates of complete aneurysm obliteration. This technique appears safe, with minimal impact on postoperative mortality and neurological outcomes.

**Supplementary Information:**

The online version contains supplementary material available at 10.1007/s10143-025-03668-x.

## Graphical abstract

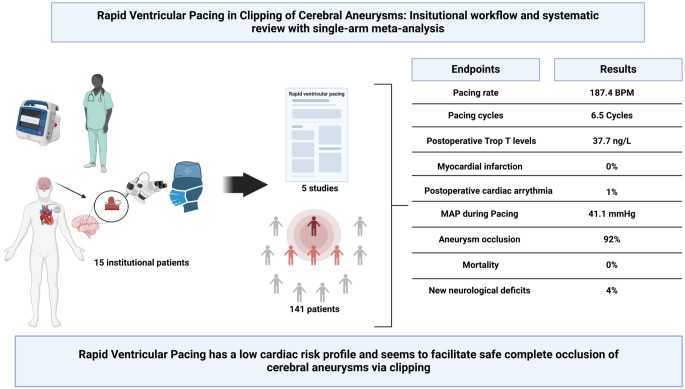

## Introduction

Most cerebral aneurysms are treated with primary clip reconstruction or endovascular coil embolization, including newer devices like flow diverters or woven endobridges (WEB). However, these newer emerging devices have not delivered the expected outcomes in terms of morbidity, mortality, and long-term occlusion rates for complex aneurysms so far [1, 2, 3, 4]. Complex and large unruptured aneurysms with wide neck, calcifications and branching vessels are challenging and sometimes need tools such as temporary clipping, adenosine-induced cardiac arrest, suction decompression, hypothermia with cardiac standstill or in very complex cases bypass techniques to potentially reduce morbidity and mortality [5, 6, 7, 8, 9, 10]. Rapid ventricular pacing (RVP) is a method used in dedicated vascular neurosurgical centers, particularly for the clipping of complex cerebral aneurysms [12, 13]. By inducing temporary hypotension through rapid stimulation of the right ventricle, RVP allows surgeons to soften the aneurysm sac and facilitate safer clip application, especially in cases where proximal vessel control is limited​​ [12, 13].

However, the evidence supporting the use of RVP remains limited to a few small-scale single-center studies [14]. This current evidence makes it difficult to generalize findings, particularly regarding the safety of the technique. Although RVP has shown promise in facilitating aneurysm clipping without significant complications, concerns remain about postoperative cardiac events. For instance, transient arrhythmias and minor elevations in cardiac troponin levels have been reported, though these usually resolve without long-term effects [14]​​​.

Against this backdrop, there is a clear need for a systematic review to consolidate existing evidence and assess both the risks and benefits more comprehensively. Furthermore, the present study also provides institutional experience and a structured workflow.

## Methods

### Institutional workflow

**Fig. 1:**
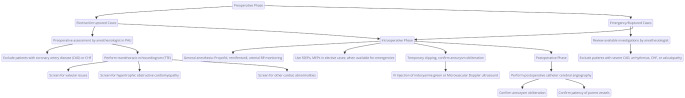
Institutional workflow to identify eligible patients for rapid ventricular pacing

RVP was implemented at the institution in January 2023, with the case series covering January 2023 to December 2024. Fifty-six cerebral aneurysms clipping procedures were performed in this time period. Institutional use of RVP is limited to complex aneurysms wide neck, calcifications, branching vessels, and limited proximal control. The present institution does not use any other methods to reduce blood flow (e.g., adenosine or hypothermia). The institutional workflow stratifies patients into elective (unruptured aneurysms) and emergency (ruptured aneurysms) cases. For unruptured cases, preoperative anesthesiological assessment included ruling out coronary artery disease and heart failure, with transthoracic echocardiograms (TTE) screening for valvular stenosis or cardiomyopathy contraindications. In emergency cases, preoperative investigations were reviewed for RVP suitability. Intraoperative management involved fellowship-trained anesthesiologists using general anesthesia, endotracheal intubation, and arterial line monitoring. Anesthesia was maintained with propofol and remifentanil, ensuring normothermia (36–37 °C). Neurophysiologic monitoring was employed for elective and available emergency cases. Indocyanine green angiography confirmed aneurysm obliteration. Postoperatively, extubation occurred if appropriate, and troponin T levels were checked if cardiac signs emerged during intensive care unit monitoring over 24 h. All patients underwent computed tomography on the first postoperative day and digital subtraction angiography during hospitalization. Figure 1 illustrates this workflow.

### Rapid ventricular pacing procedure

All patients received invasive arterial pressure monitoring after anesthesia induction (propofol remifentanil), and a central venous catheter with a pacing wire was inserted using ultrasound guidance. Correct placement of the pacing catheter was confirmed via ECG monitoring.

In the operating room, patients were positioned, and the aneurysm was prepared microsurgically with Zeiss Kinevo 900 (Carl Zeiss AG, Jena, Germany). Intraoperative indocyanine green (ICG) angiography was used to visualize the aneurysm and blood vessels. Rapid ventricular pacing (RVP) was initiated for up to 60 s as needed, with pacing rates adjusted based on patient risk factors and aneurysm response. The target middle arterial pressure (MAP) was between 40 and 50 mm Hg. Repeated RVP was used if required. Obliteration was intraoperatively assessed with ICG and all patients routinely underwent postoperative DSA within 7–10 days after the surgery.

### Search strategy

The systematic review followed PRISMA (Preferred Reporting Items for Systematic Reviews and Meta-Analyses) guidelines from the Cochrane Collaboration (PRISMA checklist in Supplementary Data 1) [15, 16]. Two reviewers independently searched PubMed, Cochrane, and Google Scholar to evaluate the efficacy] and safety of techniques improving surgical outcomes in aneurysm clipping. Key medical subject headings included “Asystole,” “cardiac arrest,” “aneurysm,” “intracranial,” “brain,” “neurovascular,” “clipping,” “subarachnoid hemorrhage,” “cardiac standstill,” “hypotension,” “flow arrest,” and “rapid ventricular pacing.” References of included studies and reviews were also analyzed for comprehensive coverage.

### Inclusion criteria

The inclusion criteria comprised: (1) patients undergoing RVP-induced cardiac arrest during cerebral aneurysm surgery; (2) randomized or non-randomized trials, as well as case series with at least five patients; and (3) studies published in the English language. The exclusion criteria were: (1) studies not available in English; (2) studies lacking relevant clinical outcomes; (3) studies involving populations outside the research scope; (4) conference abstracts, editorials, or comments; and (5) case series with fewer than five cases or studies conducted on nonhuman subjects.

### Data extraction and statistical methods

The following endpoints were defined and extracted if feasible: (1) mean applied pacing rate, (2) mean applied pacing cycles, (3) Mean arterial pressure (MAP) during pacing, (4) postoperative cardiac arrhythmia, (5) postoperative myocardial infarction, (6) postoperative Troponin T levels, (7) postoperative new neurological deficits, (8) complete aneurysm occlusion, and (9) mortality. All data from the included studies were statistically analyzed using R version 4.3.1 for proportion analysis with inclusion of weight of individual studies. Data heterogeneity was assessed using the I² statistic, with heterogeneity considered high when I² > 50% [17]. The R package *Meta* was used for analysis [18].

### Risk of bias assessment

The risk of bias in the included studies was evaluated by two independent authors (MV, JW) using the Cochrane Collaboration’s Risk of Bias in Non-Randomized Studies of Interventions (ROBINS-I) tool [19].

## Results

### Institutional series

The Table 1 outlines fifteen patients with treated for sixteen cerebral aneurysms, with eleven female patients and four male patients, aged between 19 and 69 years. Aneurysm locations varied, including the anterior cerebral artery, middle cerebral artery (MCA)-bifurcation, anterior communicating (Acom), posterior communicating artery (Pcom) and posterior inferior cerebellar artery (PICA) with sizes ranging from 4 mm to 31 mm. 10 patients had unruptured aneurysms, and the remaining patients had ruptured aneurysms, with two having AVMs with flow-related aneurysms. In one of the AVM patients with flow-related aneurysms, two aneurysms were clipped in one operation. At discharge, the modified Rankin Scale (mRS) scores were 0 for eight patients and 1 for the remaining seven, indicating minimal to no disability. Neurological functioning remained stable for all patients and no new neurological deficits occured. There were no instances of arrhythmia, myocardial infarction, or RVP-associated brain infarctions. Aneurysm occlusion was complete in fifteen of sixteen cerebral aneurysms (93.8%), with one case showing incomplete occlusion with a small neck remnant.

**Table 1:** Characteristics of institutionally treated patients

Age	Sex	Aneurysm location/size	Aneurysm status	mRS at discharge	Neurological functioning	Arrhythmia	Myocardial infarction	Brain infarction	Aneurysm occlusion
59	female	Mediabifurcation left (5 mm)	ruptured	mRS 1	No new deficit	No arrhythmia	No myocardial infarction	No RVP-associated infarction	Complete aneurysm occlusion
47	female	ICA (8 mm) & M2 (6 mm)	unruptured	mRS 0	No new deficit,	No arrhythmia	No myocardial infarction	No RVP-associated infarction	Complete aneurysm occlusion
60	female	Mediabifurcation left (7 mm) & PCom (4 mm)	unruptured	mRS 0	No new deficit	No arrhythmia	No myocardial infarction	No RVP-associated infarction	Complete aneurysm occlusion
39	female	M2 left (31 mm)	unruptured	mRS 0	No new deficit	No arrhythmia	No myocardial infarction	No RVP-associated infarction	Complete aneurysm occlusion
44	female	Pcom left (9 mm, recurrence)	Recurrence of coiled and previously ruptured aneurysm	mRS 1	No new deficit	No arrhythmia	No myocardial infarction	No RVP-associated infarction	Complete aneurysm occlusion
43	female	Pcom left (6 mm)	Unruptured (Oculomotor nerve palsy)	mRS 1	No new deficit, Regredient palsy of third nerve	No arrhythmia	No myocardial infarction	No RVP-associated infarction	Complete aneurysm occlusion
56	male	Acom left (13 mm)	Unruptured	mRS 0	No new deficit	No arrhythmia	No myocardial infarction	No RVP-associated infarction	Incomplete aneurysm occlusion (small neck remnant)
59	male	Pericallosal artery right (10 mm)	Ruptured	mRS 1	No new deficit, Regredient palsy of third nerve	No arrhythmia	No myocardial infarction	No RVP-associated infarction	Complete aneurysm occlusion
69	female	ICA right (25 mm)	Unruptured with bitemporal hemianopsia	mRS 1	No new deficit	No arrhythmia	No myocardial infarction	No RVP-associated infarction	Complete aneurysm occlusion
19	female	MCA trifurcation right (15 mm)	Unruptured	mRS 0	No new deficit	No arrhythmia	No myocardial infarction	No RVP-associated infarction	Complete aneurysm occlusion
68	female	Pcom right (9 mm)	Unruptured and previously treated with Flowdiverter	mRS 0	No new deficit	No arrhythmia	No myocardial infarction	No RVP-associated infarction	Complete aneurysm occlusion
40	male	Acom (16 mm)	Ruptured	mRS 1	No new deficit	No arrhythmia	No myocardial infarction	No RVP-associated infarction	Complete aneurysm occlusion
66	male	AVM (Spetzler-Martin grade 1) with two flow-related A2 aneurysms (20 & 12 mm)	Unruptured	mRS 0	No new deficit	No arrhythmia	No myocardial infarction	No RVP-associated infarction	Complete aneurysms occlusions
49	female	AVM (Spetzler-Martin grade 1) with flow-related PICA aneurysm left (5 mm)	Ruptured	mRS 1	No new deficit	No arrhythmia	No myocardial infarction	No RVP-associated infarction	Complete aneurysms occlusions
51	female	MCA left (5 mm)	Unruptured	mRS 0	No new deficit	No arrhythmia	No myocardial infarction	No RVP-associated infarction	Complete aneurysms occlusions

### Systematic review

**Fig. 2:**
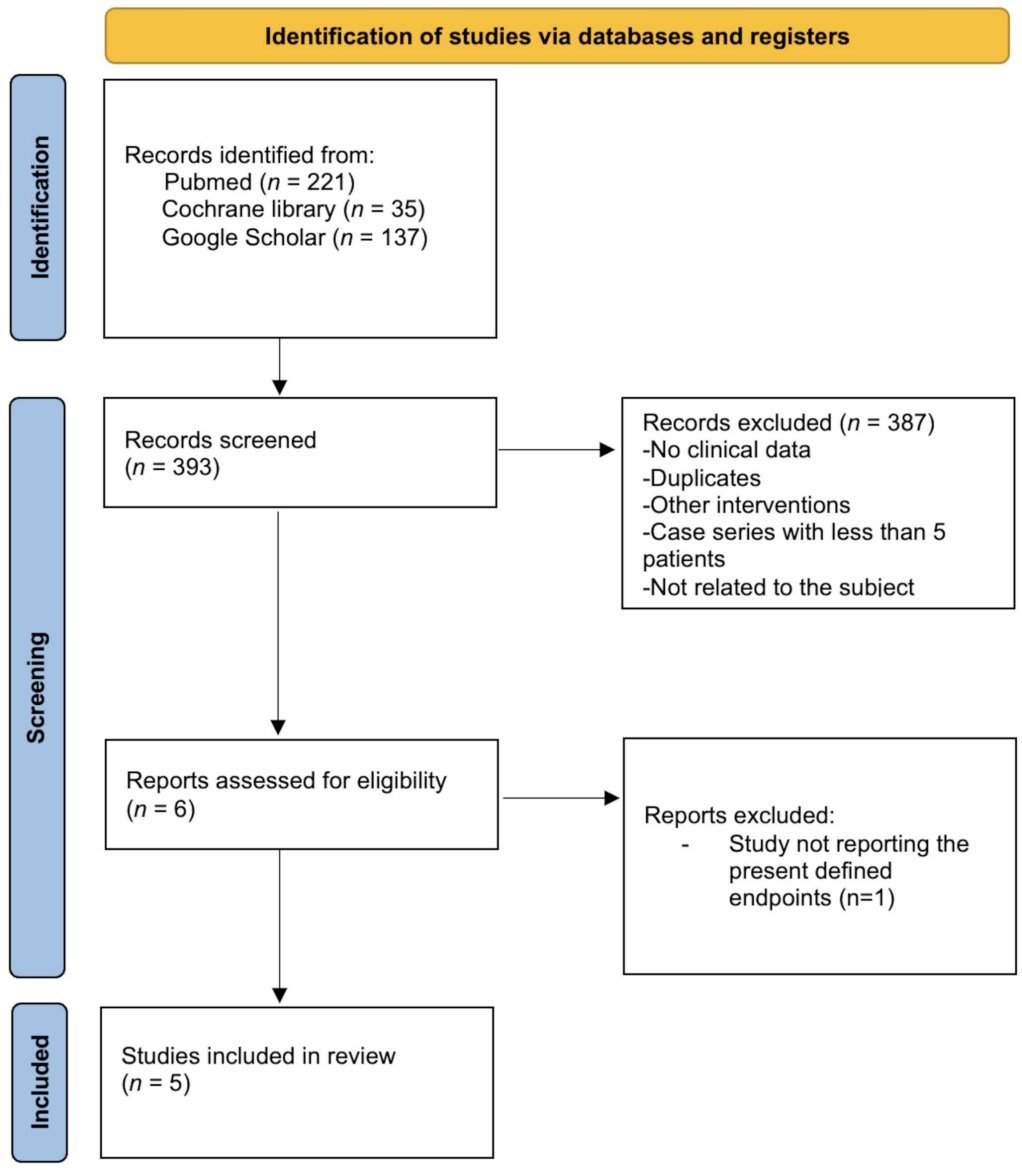
PRISMA Flow-Chart illustrating the literature search workflow

A total of 393 records were identified through PubMed (*n* = 221), Cochrane Library (*n* = 35), and Google Scholar (*n* = 137) for studies evaluating rapid ventricular pacing (RVP) in cerebral aneurysm surgery. After screening, 387 records were excluded for reasons such as lack of clinical data, duplication, other interventions, small case series, or irrelevance. Ultimately, 5 studies were included in the review, following the exclusion of 1 study that did not report the predefined endpoints (see Fig. 2) [14, 19, 20, 21, 22].

Table 2 describes the included five studies on RVP during cerebral aneurysm surgery, including prospective and retrospective designs from Belgium, Germany, and Canada. Sample sizes range from 11 to 40 patients, with aneurysms located in various arteries (e.g., MCA, ACoA, ICA), and pacing durations typically up to 60 s.

**Table 2:** Characteristics of the included studies

Authors & Year	Study design	Country	Duration of Rapid Pacing	Sample Size of cerebral aneurysm patients (Total)	Ruptured cerebral aneurysm)	Unruptured cerebral aneurysms	Aneurysm location (No.)	Aneurysm size	Mean age	Sex (female/male ratio)
Saldien et al., 2012 [20]	Prospective	Belgium	40 s	11	3	8	- SCA (1) - MCA (2) - A1 Segment (4) - ACoA (1) - PCoA (1) - Ophthalmic Artery (1) - AchA (1) - ICA (1)	NA	- 40.7	1.2:1
Konczalla et al., 2017 [21]	Prospective	Germany	60 ± 25	20	-	20	- ACoA (3) - MCA (11) - PCoA (2) - ICA (3)	11.1 ± 5.5 mm (range 6–30 mm)	− 51.6	3:1
Saldien et al., 2018 [22]	Retrospective	Belgium	NA	28	8	20	- Posterior circulation (2) - AchA (2) - MCA (14) - ACoA (4) - ACoA/AchA(1) - ICA (5)	NA	- 51 (unruptured) - 56 (ruptured)	2.5:1
Grabert et al., 2021 [14]	Retrospective	Germany	Up to 60 s	17	8	9	- AICA (1) - ACoA (1) - MCA (4) - ICA (11)	19.82 ± 9.1	− 49.2 ± 4.9 (unruptured) − 56.1 ± 4.0	2.4:1
Ragulojan et al., 2024 [23]	Retrospective	Canada	Up to 60 s	40	21	19	- ACoA (11) - ICA (12) - PCoA (8) - MCA (6) - PICA (3) - SCA (3) - AchA (2) - Posterior Spinal Artery (1)	- Small (< 5 mm) (22) - Medium (5–10 mm) (16) - Large (10–25 mm (8) - Giant (> 25 mm) (1)	53 ± 12	3:1

### Hemodynamic characteristics of rapid ventricular pacing

**Fig. 3:**
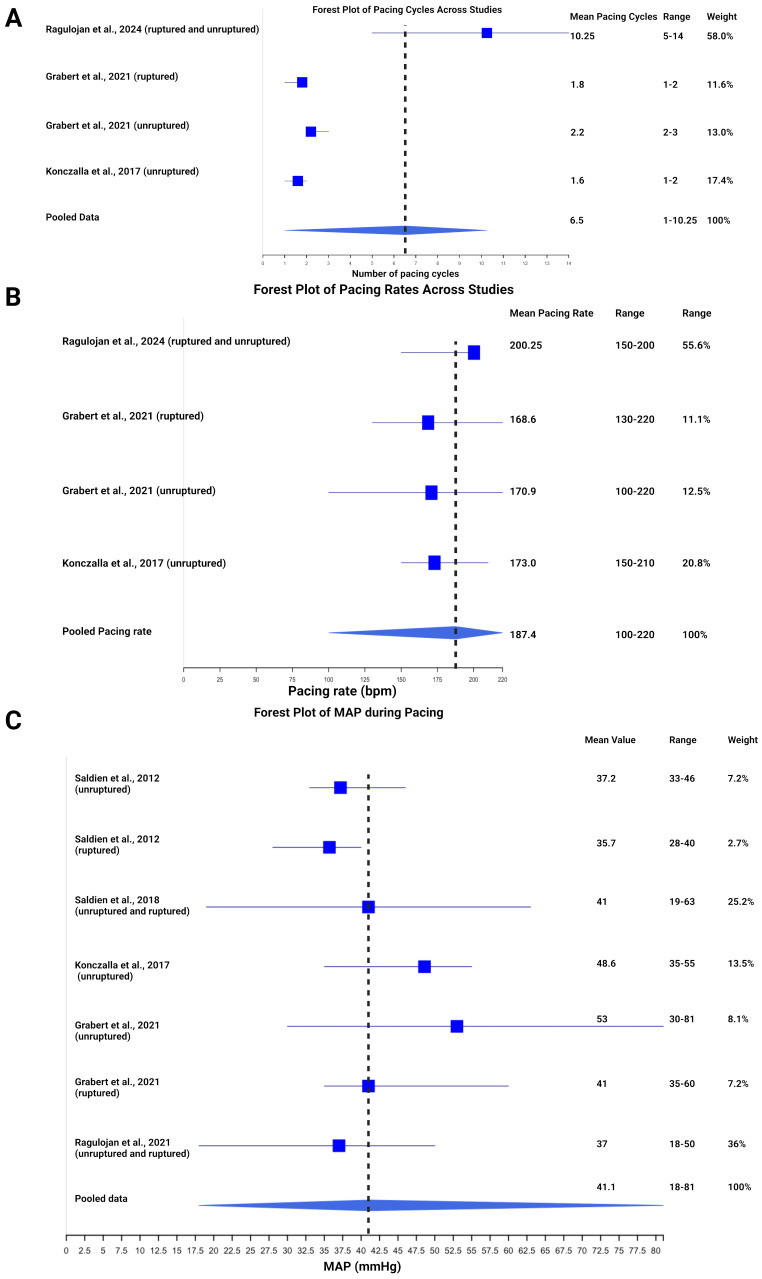
Forest plots summarizing the hemodynamic characteristics of rapid ventricular pacing (RVP) across studies. (**A**) Number of pacing cycles, (**B**) pacing rates (beats per minute), and (**C**) mean arterial pressure (MAP) during pacing, with pooled data reflecting variability in surgical protocols and patient conditions

Hemodynamic characteristics of RVP across multiple studies were analyzed. The number of pacing cycles varied across the studies, with a pooled mean of 6.5 cycles (range: 1–10.25) applied for the clipping procedures. Ragulojan et al. [23] reported the highest mean number of cycles at 10.25 (range: 5–14), contributing the largest weight (58.0%). Other studies, such as those by Grabert et al. [14], reported fewer cycles, ranging from 1.8 to 2.2, with moderate weights (11.6–13.0%, see Fig. 3A). Figure 3B illustrates pacing rates (beats per minute (bpm)), with a pooled mean rate of 187.4 bpm (range: 100–220). Ragulojan et al. [23] had the highest rate at 200.25 bpm (range: 150–200), representing 55.6% of the total weight. Other studies, including Konczalla et al. [21], reported slightly lower rates around 173 bpm, contributing moderately to the overall analysis. Figure 3C displays MAP during pacing, with a pooled MAP of 41.1 mmHg (range: 18–81). Grabert et al. [14] reported the highest MAP at 53 mmHg, while studies noted MAPs between 37 and 41 mmHg. The pooled data reflect a wide range of MAP values, highlighting variability in surgical protocols and patient conditions across studies, with pressures ranging from 18 to 81 mmHg. Grabert et al. [14] described the use of the semi-sitting position and RVP in a patient with unilateral vertebral artery occlusion and a contralateral AICA aneurysm, necessitating a strategy to manage the absence of proximal control.

### Cardiac side effects and mortality

Cardiac side effects of RVP across multiple studies, focusing on postoperative troponin T levels, mortality, arrhythmia, and myocardial infarction.

**Fig. 4:**
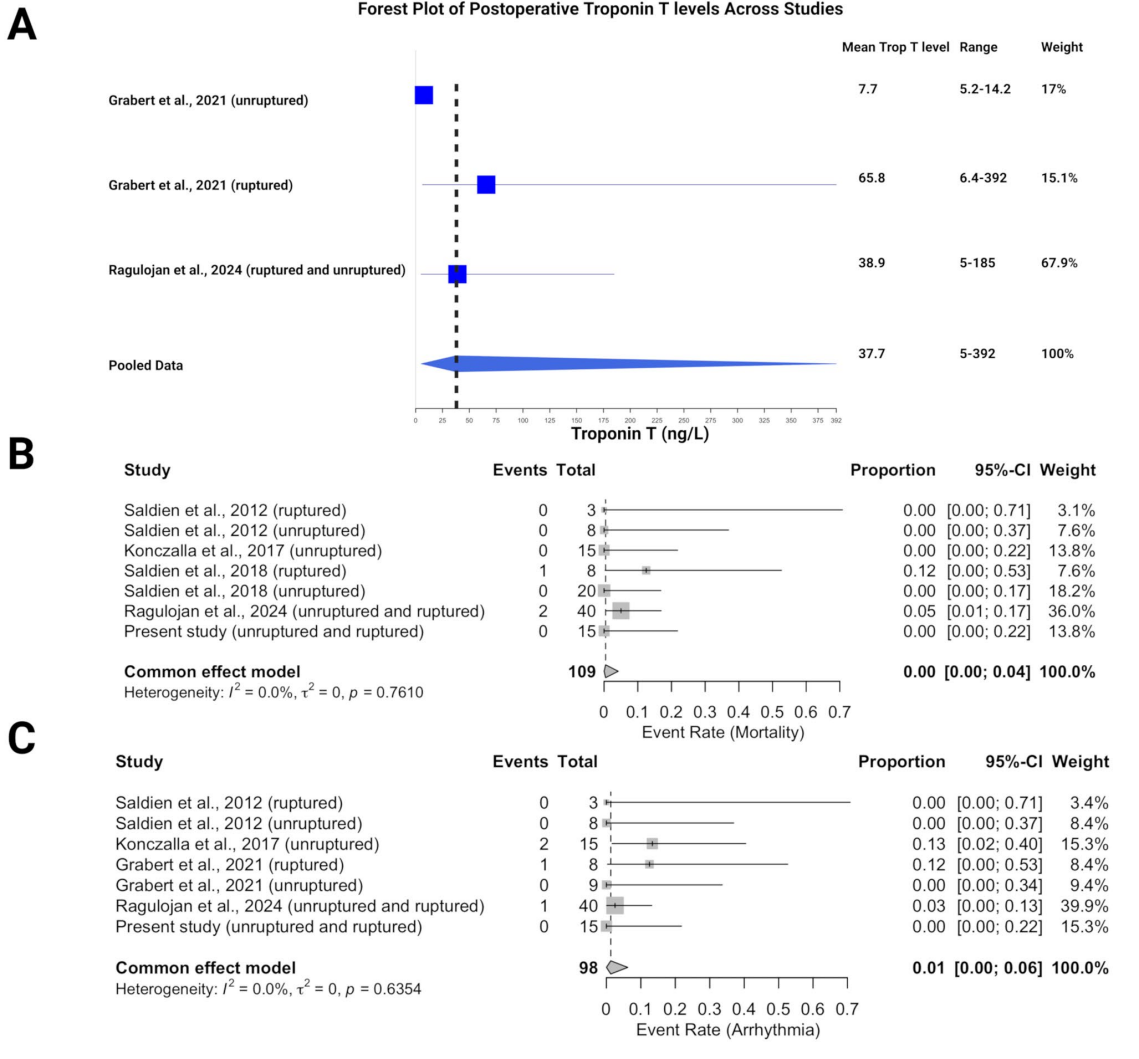
Forest plots summarizing cardiac side effects of rapid ventricular pacing (RVP) across studies. (**A**) Postoperative troponin T levels, (**B**) event rates for mortality, and (**C**) event rates for arrhythmia, with pooled data showing low rates of mortality and arrhythmia and no cases of myocardial infarction across the studies

In Fig. 4A, the forest plot summarizes postoperative troponin T levels (ng/L) across studies. The pooled mean troponin T level was 37.7 ng/L (range: 5–392), with the highest mean reported by Grabert et al. [14] for ruptured aneurysms at 65.8 ng/L, accounting for a weight of 15.1%. Ragulojan et al. [23] reported a mean troponin T level of 38.9 ng/L (range: 5–185), contributing the largest weight at 67.9%. Grabert et al. [14] also reported the lowest mean levels in unruptured aneurysms at 7.7 ng/L (range: 5.2–14.2).

Figure 4B presents the event rate for mortality across studies. No mortality was reported in most studies, with only Saldien et al. [22] and Ragulojan et al. [23] reporting events. The pooled event rate for mortality was 0.00 (95% CI: 0.00–0.04), indicating very low mortality associated with RVP. Heterogeneity between the studies was low, with I² = 0%.

Figure 4C depicts the event rate for arrhythmia. Saldien et al. [22] and Ragulojan et al. [23] reported some arrhythmia cases, while most studies reported none. The pooled event rate for arrhythmia was 0.01 (95% CI: 0.00–0.06). Similar to mortality, heterogeneity was minimal (I² = 0%), suggesting consistent findings across studies.

Overall, RVP appears to have a minimal impact on both mortality and arrhythmia, with moderate postoperative elevations in troponin T levels but no cases of myocardial infarction reported in any study. No myocardial infarctions were identified in the included studies.

### Aneurysm occlusion rate and neurological outcome

Aneurysm occlusion rate and the occurrence of new neurological deficits following RVP-assisted clipping of cerebral aneurysms were investigated across the studies.

**Fig. 5:**
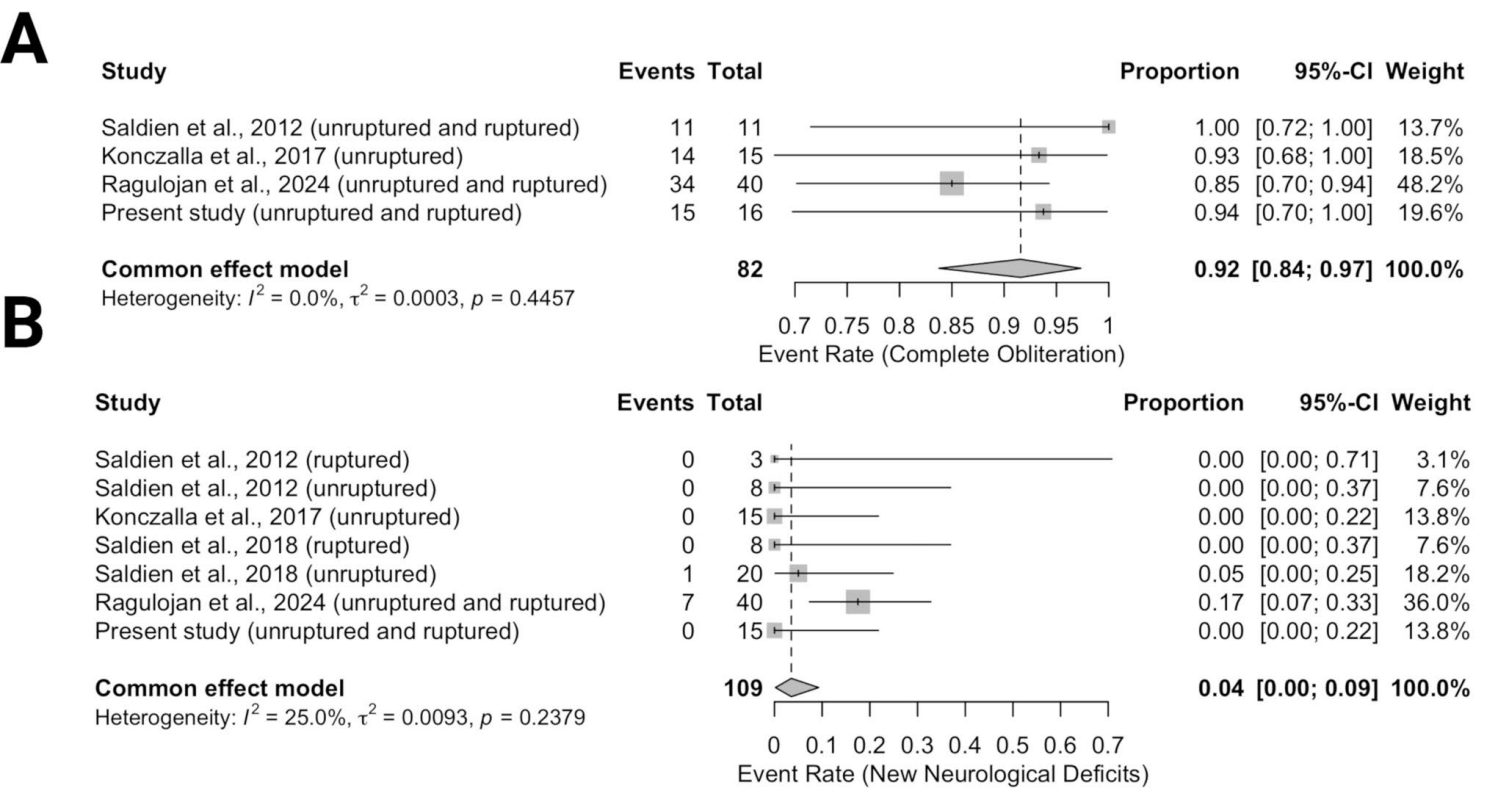
Forest plots summarizing outcomes of rapid ventricular pacing (RVP)-assisted aneurysm clipping. (A) Event rate for complete aneurysm obliteration, showing high success rates across studies, and (B) event rate for new neurological deficits, indicating a low incidence of postoperative neurological complications

Figure 5A focuses on the complete aneurysm obliteration rate. The pooled event rate for complete obliteration was 0.92 (95% CI: 0.84–0.97), indicating a high success rate for aneurysm clipping with RVP. Saldien et al. [20] reported a 100% obliteration rate (95% CI: 0.72–1.00), while Konczalla et al. [21] and the present study showed a slightly lower rate of 93% (95% CI: 0.68–1.00) and 94% (95% CI: 0.70–1.00), respectively. Ragulojan et al. [23] contributed the most weight at 60%, reporting an 85% obliteration rate (95% CI: 0.70–0.94). The heterogeneity across the studies was low (I² = 0%), suggesting consistency in the findings.

Figure 5B illustrates the event rate for new neurological deficits. The pooled event rate for new deficits was 0.04 (95% CI: 0.00–0.09), indicating a low incidence of neurological complications after RVP-assisted clipping. Ragulojan et al. [23] reported the highest event rate at 17% (95% CI: 0.07–0.33), contributing the largest weight (41.8%), while other studies, including Saldien et al. [20] and Konczalla et al. [21], reported no new neurological deficits. The heterogeneity in this analysis was also low (I² = 25%), suggesting consistent results across the studies. Ischemic brain infarctions related to RVP were not reported in the literature.

### Risk of bias assessment

The risk of bias across the studies evaluating RVP for aneurysm clipping varied. Most studies showed low bias in the selection of participants, classification of interventions, and deviations from protocols, as all adhered to well-defined criteria and standardized RVP procedures. Moderate bias was present in confounding, primarily due to the limited adjustment for factors such as aneurysm complexity and preoperative conditions. Missing data were low to moderate, with some minor gaps in secondary outcomes, like postoperative troponin fluctuations. Outcome measurement was robust in all studies, relying on tools such as MRI and troponin levels. Overall, the risk of bias was rated as low to moderate across most studies, with a few concerns in confounding and missing data handling. Supplementary Table 1 summarizes the risk of bias analysis.

## Discussion

The present institutional series and particularly the systematic review demonstrates that RVP seems to be a safe and effective technique for reducing blood flow and facilitating the microsurgical clipping of complex cerebral aneurysms. In the pooled analysis of 141 patients, the key findings are that the rates of adverse outcomes such as postoperative arrhythmias (1%) and mortality (0%) were minimal, while aneurysm obliteration was achieved in 92% of cases. These results are consistent with prior studies that also reported high obliteration rates and low incidences of postoperative complications. Importantly, our findings add to the growing body of evidence supporting flow-reducing procedures as a viable option in both ruptured and unruptured aneurysms, offering a controlled method to induce temporary hypotension and reduce intraoperative bleeding risks [24].

Previous studies have demonstrated the utility of RVP in cerebral aneurysm surgery, particularly when proximal control of the parent artery is not feasible, or in cases of intraoperative rupture [20, 21]. Saldien et al. [20] reported that RVP achieved significant reductions in blood pressure within seconds, facilitating aneurysm clipping with complete occlusion and without long-term complications​. Similarly, Konczalla et al. [21] in a prospective trial of 20 patients, observed that RVP facilitated safe aneurysm mobilization and clipping, with a mean aneurysm size of 11.1 mm and a mean RVP-induced heart rate of 173 beats per minute [21]. Hence, this method seems to be appropriate for large complex aneurysm in order to achieve complete occlusion and thereby reducing the risk of rerupture or retreatment [4, 25]. The pooled findings indicate favorable outcomes, with only minor postoperative troponin increases, consistent with our own clinical results of no cardiac events.

Notably, the pooled data builds on these findings by confirming the minimal cardiovascular risk associated with RVP. Ragulojan et al. [23] reported a transient rise in troponin levels in 42% of patients post-RVP, but no long-term cardiac events were observed​. This aligns with the synthesized data, where transient cardiac changes occurred but did not contribute to clinically relevant morbidity.

The benefit of using RVP over traditional techniques like temporary clipping or adenosine-induced cardiac arrest is suggested to lie in its predictability and control. Adenosine can cause unpredictable durations of asystole and variable hypotensive effects. In contrast, RVP allows for precise control over both the onset and duration of hypotension, thereby reducing the risk of ischemia while maintaining continuous perfusion to the brain​ [24]. Moreover, as observed in the pooled dataset, the use of intraoperative neurophysiological monitoring, including motor evoked potentials (MEPs) and somatosensory evoked potentials (SSEPs), helped mitigate the risk of neurological deficits, which occurred in only 5% of patients​. However, these deficits are predominantly described in series including aneurysmal subarachnoid hemorrhage patients who might also develop symptoms which are not directly associated with RVP-assisted clipping.

This is further supported by a pilot study by Saldien et al. [26] which explored the real-time effects of RVP on cerebral blood flow (CBF) and oxygenation. The study found that RVP-induced hypotension resulted in a reversible reduction in CBF without causing cerebral ischemia, as indicated by stable brain tissue oxygenation levels post-RVP. Our institutional workflow, which includes close hemodynamic monitoring and the use of advanced dual imaging techniques for neurovascular pathologies like intraoperative indocyanine green angiography and hyperspectral imaging combines both evaluation of perfusion with complete aneurysm obliteration and also monitoring for risk of ischemic complications [27]. During dissection and clip replacement of giant aneurysms, clip slippage can lead to perforation of the aneurysm sac. Lowered MAP via RVP can facilitate successful clip re-placement in a clear surgical field (see video 1).

Despite these promising findings, the limited sample size of the included studies remains a challenge. Many studies, including ours, have been restricted to small patient cohorts, with sample sizes ranging from 6 to 40 patients per series​. The heterogeneity of study populations (e.g., ruptured and unruptured cases in one cohort) and variations in institutional practices also complicate direct comparisons across studies. However, the overall consensus from available literature, including our systematic review, supports the efficacy and safety of RVP in complex aneurysm surgery, particularly in centers with high surgical and anesthesiological expertise.

## Conclusions

In conclusion, RVP is an effective adjunct for complex aneurysm clipping, offering controlled hypotension with a low risk of cardiac or neurological complications. Future larger, multicenter trials are necessary to further refine patient selection criteria and optimize procedural protocols, thereby facilitating wider adoption of this technique in neurovascular surgery.

## Electronic supplementary material

Below is the link to the electronic supplementary material.

Supplementary Material 1

Supplementary Material 2

Supplementary Material 3

Video 1: Illustrative case of giant right-sided ICA aneurysm which has been clipped with rapid ventricular pacing and dual neurovascular imaging via ICG-angiography and hyperspectral imaging

## Data Availability

No datasets were generated or analysed during the current study.
