# Supplementary material for: Rapid ventricular pacing in cerebral aneurysm clipping: institutional workflow, systematic review, and single-arm meta-analysis
Source: Neurosurg Rev. 2025 Jun 11;48(1):501. doi: 10.1007/s10143-025-03668-x (PMC12152091; doi:10.1007/s10143-025-03668-x)
Supplement: Supplementary file 1 — Supplementary Material 1 [file 10143_2025_3668_MOESM1_ESM.docx]

| **Section** | **Item** | **Description** | **Page** |
| --- | --- | --- | --- |
| **Title** | Title | The title identifies the report as a systematic review and meta-analysis of rapid ventricular pacing in cerebral aneurysm clipping. | 1 |
| **Abstract** | Structured Summary | The abstract provides a summary covering objectives, methods, results, conclusions, and key terms such as “rapid ventricular pacing,” “aneurysm,” and “meta-analysis.” | 2 |
| **Introduction** | Rationale | Background explains the clinical rationale for RVP, discussing the limited evidence base and potential safety concerns that prompted this systematic review and meta-analysis. | 4 |
|  | Objectives | Clearly specified objectives focused on assessing the efficacy and safety of RVP in achieving outcomes such as aneurysm obliteration, arrhythmia incidence, and mortality. | 4-5 |
| **Methods** | Eligibility Criteria | Defines eligibility criteria: includes patients undergoing RVP-assisted aneurysm clipping (ruptured or unruptured), only studies with at least five patients, and those published in English. | 5-6 |
|  | Information Sources | Databases searched include PubMed, Cochrane Library, and Google Scholar. The search period covers relevant studies from 2010 onward. Key search terms included “rapid ventricular pacing,” “aneurysm,” and related terms. | 5-6 |
|  | Search Strategy | Details search terms and Boolean combinations, like “aneurysm” AND “cardiac standstill” OR “flow arrest,” targeting studies related to RVP in aneurysm clipping procedures. | 6 |
|  | Selection Process | Study selection conducted by two independent reviewers with consensus on final inclusion criteria. A PRISMA flowchart illustrates the process. | 7 |
|  | Data Collection Process | Data extracted includes primary variables like pacing rate, mean arterial pressure (MAP), and postoperative outcomes, using standardized extraction forms. Each study's data consistency was cross-verified. | 7 |
|  | Data Items | Key data items include pacing cycles, MAP during pacing, aneurysm obliteration rate, postoperative arrhythmia, troponin T levels, and neurological outcomes. | 7 |
|  | Study Risk of Bias Assessment | Uses the ROBINS-I tool for assessing risk of bias, covering domains such as participant selection, confounding factors, and accuracy in outcome measurement. | 8 |
|  | Effect Measures | Effect measures include pooled proportions for outcomes like mortality, arrhythmia, aneurysm obliteration, and confidence intervals (CIs) for each outcome. | 7 |
|  | Synthesis of Results | Describes synthesis approach, using a common effect model to pool data and I² statistics to assess heterogeneity for each major outcome. | 7 |
|  | Risk of Bias Across Studies | Overall assessment of bias across studies, indicating low bias in outcome measurement and intervention application, with some moderate risk due to confounding variables. | 8 |
| **Results** | Study Selection | PRISMA flowchart shows records identified, screened, and included, with exclusions for studies not meeting clinical outcome criteria. | 9 |
|  | Study Characteristics | Summarizes study characteristics, including sample sizes, aneurysm locations, pacing duration, and MAP variations. | 9-10 |
|  | Results of Individual Studies | Individual study results on RVP outcomes like pacing rate, MAP, aneurysm obliteration rate, arrhythmia, and troponin T levels, illustrated with forest plots and summaries. | 10-12 |
|  | Synthesis of Results | Forest plots and pooled data provide a comprehensive view of primary outcomes, including mortality, arrhythmia, troponin T levels, and aneurysm obliteration. | 10-12 |
|  | Risk of Bias in Studies | Summarizes levels of bias, generally low to moderate across studies with attention to potential confounding and outcome measurement quality. | 13 |
| **Discussion** | Summary of Evidence | Summarizes main findings, indicating that RVP supports safe aneurysm obliteration with low risk of cardiac and neurological complications, adding to the safety profile of RVP in aneurysm surgeries. | 14-16 |
|  | Limitations | Limitations discussed include small sample sizes, variability in institutional protocols, and heterogeneity in study populations, which may affect generalizability. | 16 |
|  | Conclusions | Concludes that RVP is safe and effective, emphasizing the need for larger multicenter trials to refine patient selection and protocols. | 16 |
| **Funding** | Funding | Notes that no external funding was received for the study. | 17 |
| **Acknowledgments** | Contributions | Acknowledges technical contribution from BioRender | 16 |
