# Supplementary material for: Rapid ventricular pacing in cerebral aneurysm clipping: institutional workflow, systematic review, and single-arm meta-analysis
Source: Neurosurg Rev. 2025 Jun 11;48(1):501. doi: 10.1007/s10143-025-03668-x (PMC12152091; doi:10.1007/s10143-025-03668-x)
Supplement: Supplementary file 2 — Supplementary Material 2 [file 10143_2025_3668_MOESM2_ESM.docx]

| **Table 3.** Risk of Bias assessment | | | | | | | | |
| --- | --- | --- | --- | --- | --- | --- | --- | --- |
| **Study** | **Bias due to Confounding** | **Bias in Selection of Participants** | **Bias in Classification of Interventions** | **Bias Due to Deviations from Intended Interventions** | **Bias Due to Missing Data** | **Bias in Measurement of Outcomes** | **Bias in Selection of the Reported Result** | **Overall Risk of Bias** |
| **Saldien et al.**^20^ | **Moderate**: Confounders like aneurysm severity not fully adjusted. | **Low**: Clear inclusion criteria, all participants met surgical eligibility requirements. | **Low**: Standard RVP procedure used in all patients. | **Low**: No deviations from intended RVP protocol reported. | **Low**: No significant missing data, all key outcomes reported. | **Low**: Direct measurement of intraoperative hemodynamics, post-op complications. | **Low**: All outcomes of interest were reported. | **Low to Moderate** |
| **Konczalla et al.** ^21^ | **Moderate**: Some variability in aneurysm complexity and size, which could affect results. | **Low**: Prospective selection, based on clear aneurysm complexity criteria. | **Low**: Standardized intervention with precise pacing thresholds followed. | **Low**: No deviations from RVP protocol across patients. | **Moderate**: Some minor missing data on secondary outcomes (e.g., complications). | **Low**: Robust measurement tools used (MAP, cardiac outcomes). | **Low**: All primary outcomes reported, minor complications not fully detailed. | **Moderate** |
| **Saldien et al.** ^22^ | **Moderate**: Pre-op conditions like ischemia could confound results, limited control over such factors. | **Low**: Pre-defined criteria for selecting participants (size/location of aneurysms). | **Low**: Uniform application of pacing thresholds and monitoring. | **Low**: No evidence of deviations from intervention. | **Low**: Outcome data complete, including MRI and troponin levels. | **Low**: High-quality outcome measures (MRI, troponins) used. | **Low**: Comprehensive reporting of pre-specified outcomes. | **Low** |
| **Grabert et al.**^13^ | **Moderate**: Pre-existing cardiac conditions and differences in aneurysm locations could influence outcomes. | **Low**: Participants selected appropriately based on aneurysm complexity. | **Low**: RVP protocol consistently followed for all patients. | **Low**: No significant deviations in pacing or hemodynamic targets. | **Low**: Data completeness assured, but minor events may have been underreported. | **Low**: Troponin levels and MAP changes precisely measured. | **Low**: All key outcomes reported, but minor arrhythmia data may be under-reported. | **Low to Moderate** |
| **Ragulojan et al.** ^23^ | **Moderate**: Limited adjustment for individual factors like aneurysm history. | **Low**: Clear inclusion/exclusion criteria, well-selected patients for pacing. | **Low**: Consistent application of RVP with proper anesthesia support. | **Low**: No deviations from protocol, even in emergent cases. | **Moderate**: Some missing data on troponin fluctuations post-op. | **Low**: Comprehensive hemodynamic data, troponin levels used. | **Low**: Well-documented outcomes and adverse events. | **Moderate** |
