# Supplementary material for: Rapid ventricular pacing in cerebral aneurysm clipping: institutional workflow, systematic review, and single-arm meta-analysis
Source: Neurosurg Rev. 2025 Jun 11;48(1):501. doi: 10.1007/s10143-025-03668-x (PMC12152091; doi:10.1007/s10143-025-03668-x)
Supplement: Supplementary file 3 — Supplementary Material 3 [file 10143_2025_3668_MOESM3_ESM.pdf]

Supplementary figure 1: Graphical abstract summarizing the key results of the present study

Rapid Ventricular Pacing in Clipping of Cerebral Aneurysms: Insitutional workflow and systematic review with single-arm meta-analysis

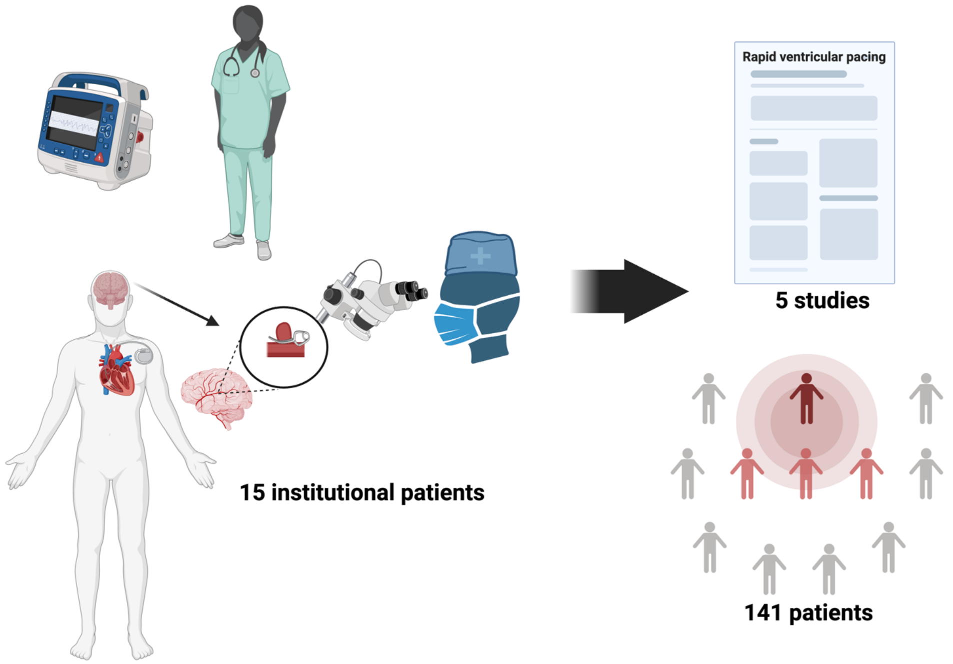

Endpoints	Results
Pacing rate	187.4 BPM
Pacing cycles	6.5 Cycles
Postoperative Trop T levels	37.7 ng/L
Myocardial infarction	0%
Postoperative cardiac arrythmia	1%
MAP during Pacing	41.1 mmHg
Aneurysm occlusion	92%
Mortality	0%
New neurological deficits	4%

Rapid Ventricular Pacing has a low cardiac risk profile and seems to facilitate safe complete occlusion of cerebral aneurysms via clipping
